# Benchmarking RNA-Seq Aligners at Base-Level and Junction Base-Level Resolution Using the *Arabidopsis thaliana* Genome

**DOI:** 10.3390/plants13050582

**Published:** 2024-02-21

**Authors:** Tallon Coxe, David J. Burks, Utkarsh Singh, Ron Mittler, Rajeev K. Azad

**Affiliations:** 1 Department of Biological Sciences and BioDiscovery Institute, College of Science, University of North Texas, 1155 Union Circle #305220, Denton, TX 76203-5017, USA; talloncoxe@my.unt.edu (T.C.); davidburks@my.unt.edu (D.J.B.); 2 Texas Academy of Mathematics and Science, University of North Texas, Denton, TX 76203, USA; utkarshsingh1302@gmail.com; 3 The Division of Plant Science and Technology, and Interdisciplinary Plant Group, College of Agriculture, Food and Natural Resources, Christopher S. Bond Life Sciences Center University of Missouri, 1201 Rollins St., Columbia, MO 65201, USA; mittlerr@missouri.edu; 4 Department of Surgery, University of Missouri School of Medicine, Columbia, MO 65212, USA; 5 Department of Mathematics, University of North Texas, Denton, TX 76203-5017, USA

**Keywords:** RNA-Seq, read alignment, alignment tools, benchmarking, *Arabidopsis thaliana*, SNPs, transcriptomics

## Abstract

The utmost goal of selecting an RNA-Seq alignment software is to perform accurate alignments with a robust algorithm, which is capable of detecting the various intricacies underlying read-mapping procedures and beyond. Most alignment software tools are typically pre-tuned with human or prokaryotic data, and therefore may not be suitable for applications to other organisms, such as plants. The rapidly growing plant RNA-Seq databases call for the assessment of the alignment tools on curated plant data, which will aid the calibration of these tools for applications to plant transcriptomic data. We therefore focused here on benchmarking RNA-Seq read alignment tools, using simulated data derived from the model organism *Arabidopsis thaliana.* We assessed the performance of five popular RNA-Seq alignment tools that are currently available, based on their usage (citation count). By introducing annotated single nucleotide polymorphisms (SNPs) from The Arabidopsis Information Resource (TAIR), we recorded alignment accuracy at both base-level and junction base-level resolutions for each alignment tool. In addition to assessing the performance of the alignment tools at their default settings, accuracies were also recorded by varying the values of numerous parameters, including the confidence threshold and the level of SNP introduction. The performances of the aligners were found consistent under various testing conditions at the base-level accuracy; however, the junction base-level assessment produced varying results depending upon the applied algorithm. At the read base-level assessment, the overall performance of the aligner STAR was superior to other aligners, with the overall accuracy reaching over 90% under different test conditions. On the other hand, at the junction base-level assessment, SubRead emerged as the most promising aligner, with an overall accuracy over 80% under most test conditions.

## 1. Introduction

The advent of next-generation sequencing (NGS) has revolutionized the fields of molecular biology and genetics. With the open accessibility of completely sequenced genomes, as well as incomplete genome assemblies in various databases, there is an unprecedented amount of genomic data now available for analyses and interpretation. In the past decade alone, the abundance of data and the significance in deciphering myriads of obscured aspects of living and extinct organisms have motivated researchers to develop methods and software tools that can interrogate the databases, often from a systems-level perspective. One such development has been the construction of splice-aware RNA-Seq alignment tools and pipelines (“aligners”), which employ diverse algorithms and methodologies to map short sequences (reads) arising from gene transcripts onto the respective reference genomes or transcriptomes. The purpose of mapping reads is to first determine their locations within the genome, and then quantify the gene expression based on read abundance at genic regions within the genome [1]. The fundamental objective of RNA-Seq aligners is to perform sensitive and accurate alignments, while sufficiently allowing for errors, maintaining minimal computational workload, and ultimately aggregating mapped reads into meaningful biological data for downstream analysis. The aligners chosen for assessment in this study were each built with their own unique purpose in mind: for instance, Subread aims to act as a general-purpose aligner of both DNA- and RNA-Seq datasets, emphasizing its ability to identify structural variations and short indels [2]. On the other hand, the alignment software BBMap places its emphasis on being splice-aware, and on its ability to align to significantly mutated genomes, accounting for long indels and >100 Kbp gene deletions [3]. 

For the purpose of benchmarking the performance of alignment software tools, we selected the model plant *Arabidopsis thaliana*. Our literature survey of benchmarking or the optimization of the alignment tools revealed a bias towards the use of mammalian, particularly human datasets. The vast amount of plant RNA-Seq data now available in the databases call for new studies to benchmark the alignment tools on plant data, which will highlight the strengths and weaknesses of the alignment tools in the plant context, providing guidelines for the proper use of these tools for plant transcriptome data analysis. This is important for the well-informed use of these tools in plant studies that can have far-reaching implications, including in agriculture, where a sustainable crop yield is critical in the face of growing populations and climate change. With a completely sequenced and well-characterized genome, *A. thaliana* provides ample resources for the benchmarking and analysis of alignment tools within a plant context. Note that there are key differences between plant and mammalian genomes (described below), and these are important distinctions since most alignment tools are pre-tuned typically for human or prokaryotic genomes. Splice-aware alignment tools for RNA-Seq are generally always optimized for human genomes. Mammalian intronic regions account for approximately 95% of the total transcribed protein-coding regions [4], while intronic and intergenic sequences comprise approximately 70% of the *Arabidopsis* genome [5]. In contrast to the mammalian intronic regions, *Arabidopsis* introns are significantly shorter in length, with ~87% of all introns not exceeding 300 bp, and <1% of all introns surpassing 1 Kbp in length [6]. For comparison, the average human intron length is approximately 5.6 Kbp, with the longest known human intron being in excess of 740 Kbp [7]. Within the context of tunable parameters in the existing alignment tools, plant and animal genome differences with respect to the introns, indels, duplications, and variant frequencies of organismal polymorphic landscapes are contributing elements that may affect alignment performance. As the default settings of most alignment tools are not tailored towards plant genomes, we rigorously tested each algorithm’s ability to align accurately to the Arabidopsis genome, using both base-level and junction base-level scoring to assess how well the alternative splicing events are deciphered by each aligner. 

The benchmarking of bioinformatic tools requires relevant well-characterized datasets, which, however, are lacking for most of these purposes, including the assessment of RNA-Seq aligners. Simulation provides an alternative route to address this problem, and here, we selected an RNA-Seq read generation tool, Polyester, which offers advantages over other RNA-Seq simulation tools through its ability to generate sequencing reads with biological replicates and specified differential expression signaling [8]. Polyester’s ability to simulate differential expressions is significant, particularly when considering that what might be an exon in one isoform of a gene may be part of an intronic region in another isoform. This process, known as alternative splicing, has been documented in prior studies featuring *Arabidopsis thaliana* to unveil differential expression patterns linked to abiotic and biotic stresses [9]. In what follows, we further elaborate on read simulations using Polyester, describe the alignment methods selected for assessment, and report and discuss the comparative assessment of these methods.

## 2. Materials and Methods

**Figure 1:**
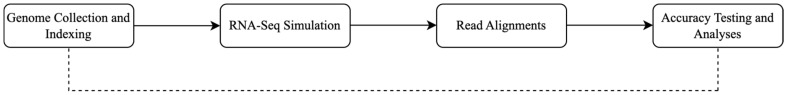
The general workflow for benchmarking alignment software tools, which includes four fundamental steps: genome collection and indexing, RNA-Seq simulation, aligners setup, and accuracy testing and analyses.

The fundamental computational workflow in most RNA-Seq pipelines usually begins with mapping sequence reads to either a reference genome or a transcriptome [10]. Typically, the mapping of reads to a reference genome involves aligning millions of short reads to the genome using an alignment algorithm. The central goal is to reconstruct the origin of each read by identifying the location in the genome that yields the optimal alignment for the read. Regardless of the program, alignment generally involves the same basic principles: building the genome index, aligning the reads, and examining the output. Our assessment pipeline consisted of four main steps: genome collection, followed by indexing that facilitates the rapid querying of reads for alignment to the genome; simulating RNA-Seq data using Polyester; performing the alignment of RNA-Seq reads using each aligner; and, finally, computing the alignment accuracy for each tool, followed by comparative assessments to highlight their strengths and weaknesses (Figure 1). 

## 3. RNA-Seq Alignment Methods

### 3.1. HISAT2

HISAT2 is the successor to TopHat2, and is designed to provide the accurate and efficient spliced alignment of RNA-Seq reads [11]. In contrast to TopHat2, HISAT2′s indexing mapping algorithm, Hierarchical Graph FM indexing (HGFM), enables it to generate multiple local, small indices for all genomic regions that comprise both the reference genome, as well as variants, in order to provide a more efficient mapping algorithm [11]. HISAT2 allows searching for local genomic regions that span multiple exons; additionally, this local alignment approach to mapping requires significantly less computing power when compared with the global indexing algorithm of TopHat2, as well as the HISAT2′s predecessor, HISAT [12]. Furthermore, HISAT2 aligns both DNA and RNA sequences using a graph Ferragina Manzini index [13], a derivation of the Burrows−Wheeler transform. This algorithm operates by indexing repeat sequences present within a genome. After a linear graph of the reference genome is produced, variants (mutations and indels) are incorporated into the index before performing an alignment. One of HISAT2′s main advantages lies in its search and storage of local indices, allowing for a fast and efficient lookup and the subsequent alignment of reads to a reference genome. By merging *k*-mers into repeat sequence indices, HISAT2 benefits from greater computational efficiency by eliminating the necessity of storing an overabundance of genome coordinates in order to identify a read’s location within its reference genome. By merging the instances in which *k*-mers have occurred into repeat sequences at least *C* times, HISAT2 ensures that reads with a high degree of occurrence within the reference genome are mapped to all known locations. However, reads containing sequences that are present *n* times (*n* ≥ *C*) will be mapped to a single repeat sequence [13]. 

### 3.2. STAR

Whereas HISAT2 operates by generating short, local indices for genomic regions comprising the reference genome for subsequent mapping, STAR’s alignment algorithm consists of a seed-searching step and a clustering/stitching/scoring step [14]. STAR’s seed-searching step involves locating a maximal mappable prefix (MMP), beginning with the first base of a read, with a “seed” being defined as a shorter part of the read that can be mapped to the genome. The algorithm systemically maps each seed in accordance with its MMP to allow for the discovery of splice junction locations within each read sequence. One major advantage of STAR is its ability to detect splice junctions in the absence of junction databases, with MMP search occurring a priori—implementing suffix arrays (SA) to obviate the need for greater computational power and reduce search time. Additionally, STAR’s MMP search is capable of detecting mismatches and indels via an extension procedure that anchors read incongruencies, as they are aligned to the reference genome. This is performed in both the forward and reverse directions of sequenced reads to facilitate improved mapping sensitivity under conditions where high error rates may be present. The extension procedure of STAR does, however, present the possibility of producing a poor genomic alignment, resulting in the chance of the algorithm finding poly-A tails, adapter sequences, or others with poor sequencing quality. After the seed searching phase, the latter step of STAR’s alignment algorithm entails the stitching together of seed alignments. This is accomplished through the clustering of these sequences based upon their “anchoring” within the genome—the anchor selection process being discriminated by a limitation on the quantity of genomic loci that the anchors are being aligned to. This process of stitching and clustering is carried out contemporaneously with the seeds of mates of paired-end RNA-Seq sequence reads in the STAR alignment, with the tracking of mate overlapping being an approach to boost alignment sensitivity. In the event that an alignment within a single genomic window is insufficient to span the entire read, STAR’s alignment algorithm searches for two or more windows to generate a chimeric read alignment, consisting of various pieces of read mapping to then be assigned to other portions of the RNA molecule. Chimeric mates may be internally aligned and used to distinguish between the points in the genome that may contain chimeric junctions, allowing for the detection of chimeric regions in the downstream analysis. The stitching of seeds in STAR follows a local-alignment scoring system, with penalties for mismatches, indels, and gaps allowed to be defined by the user. The final output is the highest scoring combinations of seed stitches generated in the initial phase.

### 3.3. Subread

Subread employs an alignment algorithm similar to that of STAR, using a “seed-and-vote” strategy. This involves the usage of multiple short reads from each read, referred to as subreads, to determine the most ideal location within the genome for mapping to occur [2]. Subread’s purportedly quick and accurate alignment algorithm has been attributed to two elements in particular by its creators: firstly, the location of subreads within the genome is determined prior to the completion of comprehensive alignment; additionally, Subread’s algorithm does not place requirements on exact individual subread mapping, nor does it enforce any constraints to map subreads within close proximity to each other. This acts as both an algorithmic shortcut to evade the computationally onerous task of read alignment, as well as a strategy to increase the alignment sensitivity. Secondly, the algorithm’s accuracy is accredited to the requirement of each subread’s inferred location within the genome to be supported by information gained from the mapping of numerous other subreads. This strategy may also help locate exon junctions, with reads containing multiple subreads being further analyzed in order to locate the mapping locations for different exons of the same gene. The mapping quality score (MQS) that Subread uses is obtained as follows:

$$MQS=100+\frac{100}{l}\left\{\sum_{i\epsilon b_{m}}\;(1-p_{i})-\sum_{i\epsilon b_{mm}}\;(1-p_{i})\right\}.$$

Here, *l* denotes read length, *p_i_* is base-calling *p*-value for each *i*th base contained in the read, *b_m_* represents the group of genome locations for matched bases, and *b_mm_* represents the group of genome locations for mismatched bases. MQS is based upon the read-length, and is a normalized value lying within 0–200. In the event that a read may be mapped to more than one location, the read’s MQS is divided by the number of these locations. 

### 3.4. BBMAP

In contrast to the aforementioned alignment tools that map seeds onto the reference genome, BBMap aligns short k-mers to the reference genome. Although BBMap may be applied for use with RNA sequencing reads, it primarily operates as a DNA aligner; thus, its compact idiosyncratic gapped alignment report (CIGAR) strings are based on DNA alignment logic with junctions being labeled as deletions. In the case of BBMap, intronic junctions and deletion variants are considered identical for the purpose of this study, whereas the other alignment algorithms considered here are RNA-based, and therefore assume that the input data is RNA—with long stretches missing in read alignments considered introns rather than indels. In the BBMap’s algorithm, prior to the processing of reads, the reference genome is indexed using a k-mer size of 13 at the default setting. While it is possible to adjust this value, shorter k-mers allow for increased sensitivity; however, this comes at the cost of rapid alignment attained using longer k-mers [15]. BBMap utilizes a sliding window approach to index generation, wherein for every k-mer present in the window, a ‘key’ is made. Each key is given a unique integer code to track their abundance within the index. BBMap’s indexing method operates by creating two arrays: sizes and sites. The genome index creation consists of three steps: 1. The occurrences of each key is recorded. 2. Next, the creation of the array sizes occurs by taking the total summation of temporary values created in the previous step, then arranging their values in sizes by ascending numerical order. 3. Lastly, the second array, sites, is created by taking the total summation of the temp array, similar to the previous step. However, the position in sites that the key must be placed in depends upon the following algorithm: for key at the starting location in the genome, call the value from sizes array at position, and replace the positional value from the sites array with the location value, and lastly, adding k + 1 incrementally to the sizes array. This indexing algorithm results in the sites array, possessing all locations of every key. The alignment algorithm begins with the creation of triplets upon each initial hit of every key that has occurred at least once in the genome; with each triplet represented in the array by the column denoting its parent key, and the row denoting its position from sites, information is obtained about its hit location in the genome. Additionally, the triplet’s site value, or the hit position within the genome, is subtracted by the offset of its related key value. This occurs to build alignments from the subset of triplet values, based on whether its values are higher or lower than the lowest value produced. The set of triplets created are then further scrutinized by a process called ‘Triplet Translation’, wherein each triplet is systematically removed from the set after all key hits have been processed. 

### 3.5. Genome Retrieval

We downloaded the latest version of the Arabidopsis genome release, TAIR10. The genome was then processed for building its index. Open-source R package, Polyester, was used to generate reads from the TAIR10 cDNA file. The reads were generated using the following default setting of Polyester:


**Read Length**

**Error Rate**

**Error Model**

**Reads per Transcript**
100 bp0.005Illumina510,000,000

### 3.6. RNA-Seq Simulation

We used the latest version of Arabidopsis cDNA data, namely TAIR10 cDNA (file: TAIR10_cdna_20101214_updated), for generating reads using Polyester. Note that reads generated from mRNA data do not contain or span intronic regions. We built a local database of synthetic Arabidopsis “intronless” reads using Polyester, with the goal to test the ability of alignment programs to account for splicing. Aligners such as HISAT2, TopHat2, and STAR use a process known as gapped alignment, which aligns reads to the full genome, allowing for gaps in the alignment to account for the presence of introns. Other popular alignment tools, such as Salmon and Kallisto, align to transcriptomes derived from cDNAs, as opposed to the traditional alignment to the reference genome that allows for the inference of isoforms based on aligned reads, which may not be possible to this extent based on the alignment to transcriptomes. For this reason, we have chosen to use alignment programs that, by their design, align solely to genomes. 

A Python script was written for the introduction of known SNPs from TAIR10 at any percentage provided by the user. Upon the input of a given FASTA file, the SNP introducer script parses through each read to identify all known variants. This is accomplished by identifying the mate location syntax of the Polyester-generated reads in order to locate variants within a given region, filtering all intronic variants through the use of the exon variant list available from TAIR10. The output of this program produces two files: a Sequence Alignment Map (SAM) file and a FASTA file. The SAM file produced by our script contains the CIGAR string for an optimal alignment, whereas the FASTA sequence file produced contains the original sequence with the inserted SNPs. After the introduction of the SNPs, the base-level score is generated with another Python script that takes the raw alignment SAM file from each aligner as its input, and compares it to the ground truth—or the optimal alignment—SAM file created by our script. This program outputs a tabulated series of values, displaying the total read bases aligned, total junction bases, total error bases, and total deletion bases, in addition to all respective CIGAR string operations. These SAM files are later utilized in the base- and junction base-level scoring for each aligner. 

### 3.7. Aligner Setup and Indexing

An Arabidopsis genome index was built for BBMap, HISAT2, STAR, and SubRead. The purpose of building an index is to allow each aligner to organize and order genomic data for more efficient querying; additionally, data processing and subsequent compression supports the computational performance for processing the queries faster. Each of the aforementioned alignment tools possesses a unique indexing algorithm; the differences lie in index-size generation, the quantity of indexes, as well as their means of locating indexes within the reference genome. 

We tested the aligners at four different parametric settings: Default, Loose, No Reference, and Loose and No Reference. We set the same parameters for each alignment software for each respective setting. For example, STAR’s alignment parameters were as follows:


**Default Parameters**

***STAR --Runthreadn 20***

**Loose Parameters**

*STAR --runThreadN 20 --outFilterMismatchNmax 999 --outFilterMismatchNoverLmax 1 --outFilterScoreMinOverLread 0 --outFilterMatchNminOverLread 0*


‘No Reference’ alignments were performed with the same parameters as ‘Default’; however, the indices of all alignment tools would be the aligned reads in the absence of the reference genome. Similarly, ‘Loose and No Reference’ alignments were performed with the parameters characterized by ‘Loose’ alignments, while maintaining the absence of a reference genome during the indexing step. This setting serves as the baseline for the bare minimum accuracy, as the aligners are essentially enforced to perform an alignment without the *A. thaliana* reference genome and generate output, regardless of the threshold or performance. All ‘Loose’ alignments were performed to produce the best alignment with minimal impedance (no cutoff) when filtering mismatch errors, or with regard to the ratio of mismatch errors to read length; thereby allowing all alignments—regardless of score value—to be outputted.

### 3.8. Performance Assessment

Each aligner was assessed for their ability to accurately align the RNA-Seq reads generated by Polyester onto the reference genome. Once the alignments of reads were completed by an aligner, a corresponding SAM file was generated. A total of 220 SAM files were generated, representing different testing conditions by all aligners assessed in this study. Another Python script was composed to calculate the base-level and junction base-level accuracy for each experimental condition, in association with each aligner. The script requires two inputs: the ground truth SAM file, and the SAM file produced by the alignment software under investigation. A series of functions were incorporated to facilitate the processing of SAM and CIGAR strings, as well as to conduct supplementary genomic analysis. This analysis aims to compare the aligner-generated SAM file with the ground truth CIGAR string derived from the corresponding optimal-alignment SAM file. Once the total read, junction, deletion, and insertion bases, as well the error bases, were computed, the performance was quantified using the accuracy metrics Recall, Precision, and F1-score, as defined below.

$$Recall=\frac{true\;positive}{true\;positive+false\;negative}$$

$$Precision=\frac{true\;positive}{true\;positive+false\;positive}$$

$$F1\;score=2*\frac{Precision*Recall}{Precision+Recall}$$

The F1-score, as a harmonic mean of Recall and Precision, summarizes the overall performance, and we, therefore, used this metric for the assessment of the overall accuracy in the read alignments produced by different RNA-Seq aligners.

Here, we placed an emphasis on gene and transcript count data, which is appropriate for RNA-Seq differential expression analysis. We observed that each aligner produced slightly varying CIGAR strings, depending on the SAM version used (v1.3 or v1.4). STAR uses v1.3, meaning that aligned portions are simply represented by the “M” object, leaving room for ambiguity as to whether a mismatch or match of a nucleotide at a given base location had truly occurred. Whereas in BBMap (v1.4), the SAM output allows for the specification of the match/mismatch status of aligned regions by splitting the M object into “=” and “X” objects, where “=” denotes nucleotides that are matched (identical), and “X” is for aligned nucleotides that are mismatched (non-identical). Confronted with the DNA-centric mapping logic of BBMap, particularly its inability to differentiate between N (intronic junctions in RNA-Seq) and D (deletions in DNA), we adopted a streamlined approach; we chose to combine N/D calls in BBMap’s score files, and based our statistical calculations upon this approach for the junction base-level analysis. Ultimately, this simplified protocol relinquishes the need for arbitrary cutoffs or secondary classifications, thereby reducing potential errors and enhancing reproducibility. Furthermore, the unified treatment of N/D calls facilitates a more transparent acknowledgment of BBMap’s inherent limitations. While aligners like BBMap still adhere to v1.3 of the SAM format, we maintained our ‘ideal’ CIGAR string within this framework for consistency in our experiment. 

To quantify the accuracy of alignment by an RNA-Seq aligner, both base-level and junction base-level accuracy scores were computed. Of the two, the base-level metric requires an individual read base to be either ‘match’, ‘mismatch’, or aligned to a gap, when aligned against the reference genome. Splice junctions, or simply junctions, are sites in the genome where the splicing of introns results in mature mRNAs. The ability of an RNA-Seq alignment algorithm to correctly identify a junction signifies its accuracy in identifying anchors and splicing signals; an anchor represents a short aligned portion over an exon–intron junction, and the splicing signal designates the binding site at which splicing factors may attach themselves in order to remove introns from mRNA [16]. 

## 4. Results

We aimed to benchmark popular RNA-Seq alignment tools using simulated data derived from the model organism *Arabidopsis thaliana*, addressing the need for the performance assessment of these tools on plant data, and to aid in their calibration for plant transcriptomic studies. We compared the performance of five widely-used RNA-Seq alignment tools: BBMap [17], STAR, TopHat2 [18], SubRead, and HISAT2, based on their citation count. We introduced annotated SNPs from The Arabidopsis Information Resource (TAIR) to evaluate alignment accuracy at both base-level and junction base-level resolution under different parameter settings. Our results indicate that the performance of the aligners was consistent at the base-level accuracy, but varied at the junction base level, depending on the algorithm. By identifying the strengths and weaknesses of each tool under varying conditions, our study provides a valuable benchmark for researchers working with plant transcriptomic data to select the most suitable alignment tool for their needs.

**Figure 2:**
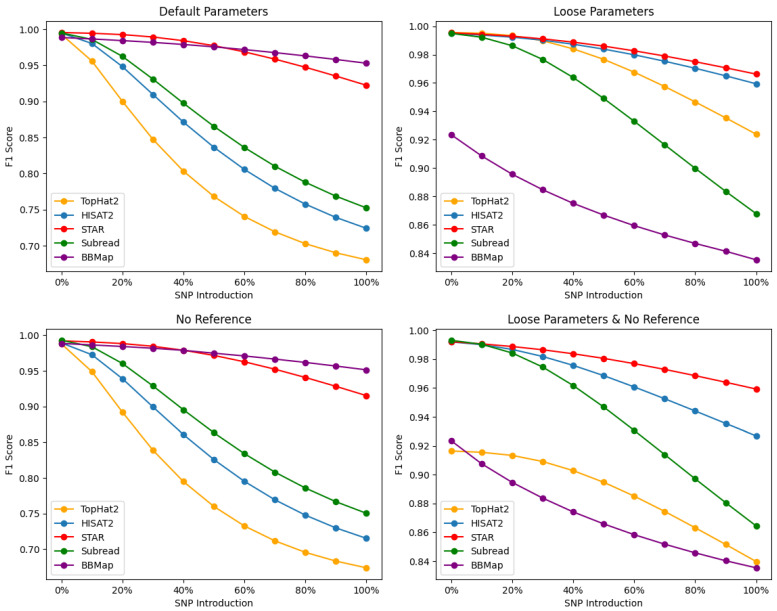
Comparative analysis of read base-level accuracy (F1-score), produced by RNA-Seq aligners at different parametric settings.

Our results, shown in Figure 2, demonstrate varying degrees of performance across all alignment tools, with STAR maintaining robust alignments irrespective of the parametric settings and SNP content. BBMap displays competitive performance to STAR, outperforming it particularly with default and no reference alignments at higher SNP levels, yet it struggles to maintain robustness once the alignment threshold is lowered, as seen with the “loose” performance (Figure 2, bottom right). Under default settings and higher SNP levels, the aligners were ranked based alignment F1-scores as follows: BBMap > STAR > SubRead > HISAT2 > TopHat2. When employing loose settings, the ranking became STAR > HISAT2 > TopHat2 > SubRead > BBMap. In the absence of a reference and at higher SNP levels, the ranking was BBMap > STAR > SubRead > HISAT2 > TopHat2. Lastly, when utilizing both loose settings and no reference, the ranking was as follows: STAR > HISAT2 > SubRead > TopHat2 > BBMap. These differences can be attributed to various factors, such as the underlying alignment algorithms, speed, sensitivity, and handling of complex RNA-Seq data.

### 4.1. Default Parameters (Read Base Level)

The F1-scores at default settings (Figure 2, top left) indicate that both BBMap and STAR consistently maintain strong performances across the entire SNP percentage range, suggesting that they are robust tools for handling plant-based datasets at varying levels of sequence divergence at read base resolutions. The superior performance of STAR can be attributed to its efficient and sensitive algorithm for detecting both splice junctions and novel splicing events, even in the presence of SNPs and other genomic variations. However, at >50% SNP introduction, BBMap reveals itself as a contender to STAR’s alignments—with a lower drop-off above this threshold and more consistent accuracy. BBMap’s robust alignments above 50% SNP introduction demonstrate its adaptability to high polymorphic variation and error tolerance at default settings. 

SubRead exhibits fair performance, particularly at lower SNP percentages, although its F1-scores decrease as the SNP percentage increases—suggesting that SubRead may be less efficient in handling datasets with a high degree of variation compared to STAR. SubRead’s performance could be affected by its emphasis on detecting structural variations and short indels in both DNA and RNA-Seq datasets. TopHat2, on the other hand, demonstrates the lowest overall performance among the five tools, particularly at higher SNP percentages. This aligner relies on the Bowtie2 aligner for read alignment, which may limit its ability to handle complex RNA-Seq data and novel splice junctions. The lower performance of TopHat2 might also be attributed to its two-step alignment process, which can be less efficient in detecting splice junctions when compared to the algorithms employed by STAR and HISAT2.

Comparing TopHat2 and HISAT2, the latter demonstrates higher F1-scores across the entire SNP percentage range, indicating improved performance over its predecessor. HISAT2 was developed to address some limitations of TopHat2, and its superior performance can be attributed to several factors. These include an improved algorithm for splice-aware alignment, enhanced speed, and increased sensitivity in detecting splice junctions. The higher F1-scores of HISAT2 suggest that it is better suited for handling complex RNA-Seq data, compared to TopHat2.

### 4.2. No Reference Parameters (Read Base Level)

Upon analyzing the F1-scores at the No Reference alignment setting (Figure 2, bottom left), BBMap and STAR continue to demonstrate strong performance, albeit with F1-scores slightly lower than those observed at default alignment settings. This observation indicates that BBMap and STAR remain robust even in the absence of reference annotation, which may be particularly relevant for studies involving organisms with incomplete or unannotated genomes. SubRead also shows markedly similar performance here (compared to Default setting), with F1-scores steadily declining as the SNP percentage increases. This trend further supports that SubRead may be less efficient in handling datasets with high levels of variation compared to BBMap and STAR. SubRead’s performance could be affected by its emphasis on detecting structural variations and short indels in both DNA and RNA-Seq datasets. TopHat2 displays the lowest overall performance among the five tools, particularly at higher SNP percentages.

Comparing TopHat2 and HISAT2 in the No Reference alignment setting, HISAT2 demonstrates higher F1-scores across the entire SNP percentage range, indicating an improved performance over its predecessor.

### 4.3. Loose Parameters (Read Base Level)

In the Loose alignment setting (Figure 2, top right), STAR demonstrates the highest overall performance, maintaining its robustness even with increased stringency. Similarly, HISAT2 exhibits a strong performance in the Loose alignment setting, comparable to STAR, with F1-scores only declining slightly as the SNP percentage increases. This trend suggests that HISAT2 is efficient in handling plant data with varying levels of genomic variation, even when loose parameters are applied, although it may be marginally less effective than STAR. SubRead’s performance at the Loose alignment setting declines considerably as the SNP percentage increases. This pattern suggests that SubRead may be less efficient in handling datasets with high levels of variation compared to STAR and HISAT2. SubRead’s performance could be affected by its emphasis on detecting structural variations and short indels in RNA-Seq datasets. Interestingly, TopHat2 generated relatively high F1-scores in the Loose alignment setting, with only a slight decline as the SNP percentage increased. However, it is worth noting that TopHat2’s performance at higher SNP percentages remains lower than that of STAR and HISAT2. Comparing TopHat2 and HISAT2 in the Loose alignment setting, HISAT2 produced higher F1-scores across the entire SNP percentage range, further indicating an improved performance over its predecessor. Lastly, BBMap produced the least accurate alignments at the Loose setting for read-base resolution. This could indicate that BBMap’s algorithm is highly sensitive to mismatches and indels, which is generally an advantage for the accurate (exact or nearly exact) mapping of reads. However, sequence data characterized by a high degree of variation may be unfavorable for Loose alignments produced by BBMap. It is important to note that Loose alignment parameters slightly vary for each tool’s algorithm, meaning that each aligner prioritizes accuracy and error correction differently under their respective settings.

### 4.4. Loose and No Reference Parameters (Read Base Level)

In the Loose and No Reference alignment setting, STAR demonstrated the highest overall performance, consistently maintaining robust F1-scores across all SNP percentages (Figure 2, bottom right). HISAT2 also exhibited strong performance in the Loose and No Reference alignment setting, with F1-scores declining slightly as the SNP percentage increases. This trend suggests that HISAT2 efficiently handles datasets with varying levels of genomic variation, even without a reference genome, albeit less so than STAR. SubRead’s performance in the Loose and No Reference alignment setting declines considerably as the SNP percentage increases, which suggests that SubRead may be less efficient in handling datasets with high levels of variation without a reference genome compared to STAR and HISAT2. 

TopHat2 yielded the second lowest F1-scores in the Loose and No Reference alignment setting across the entire SNP percentage range, indicating a lower overall performance compared to the other alignment tools (excluding BBMAP). Moreover, TopHat2 has a significantly lower performance than the other aligner tools with its F1-score declining by nearly 10%, even at 0% SNP, compared to its performance at other parametric settings. Comparing TopHat2 and HISAT2 in the Loose and No Reference alignment setting, HISAT2 demonstrated higher F1-scores across the entire SNP percentage range, indicating its superior performance over its predecessor. Lastly, BBMap exhibited the least accurate alignments overall in the absence of a reference and Loose parameters—gradually declining in accuracy as SNPs were introduced. We recognize each algorithm’s ability to handle sequence data differently, which may affect performance based on their distinctive indexing techniques and other contributing factors. 

### 4.5. Read Base Alignment Summary

The comprehensive analysis of read base alignment accuracy across different SNP percentages and parameter settings reveals nuanced performances by each alignment tool. STAR’s algorithm consistently exhibited a superior overall performance, sustaining robust F1-scores across the entire range of SNP percentages, affirming its robustness and efficiency in handling various levels of sequence divergence using the *A. thaliana* genome. Notably, BBMap emerged as a strong competitor, particularly under default parameter settings. Its performance was comparable to STAR, especially at higher SNP introduction levels, where it displayed less drop-off in accuracy, indicating its adaptability and error-tolerance in handling polymorphic variations. This suggests that BBMap’s algorithm is particularly resilient to higher amounts of SNPs in reads, potentially making it a preferred choice in genomic studies with substantial variations. 

SubRead and HISAT2 also showed commendable performances in certain settings. HISAT2 proved to be a viable alternative to STAR, especially in the absence of a reference genome and under loose parameters, while SubRead’s strength seemed to wane as SNP percentages increased. TopHat2, while being the least performant overall, might still offer some utility in specific contexts, despite its lower efficiency in aligning reads in the absence of a reference genome and under Loose settings.

Under Loose parameters, BBMap’s performance dipped, which may reflect its stringent approach to alignment, even when parameters are relaxed. It suggests that while BBMap is highly accurate, its sensitivity to mismatches and indels becomes a limiting factor when handling datasets characterized by a high degree of sequence variation. The algorithm’s stringent error correction and emphasis on accuracy over speed could be a reason for its lower performance in the Loose setting. Furthermore, BBMap’s least accurate alignments in the absence of a reference and under Loose parameters may indicate that its algorithm is optimized for different conditions, and may require a careful adjustment of settings to achieve the best results.

Our analysis of the alignment tools and parameters highlights the importance of selecting the most appropriate RNA-Seq alignment tool, based on the specific research context, dataset complexity, and the availability of a reference transcriptome. For aligning RNA-Seq reads against respective plant-based genomes, STAR remains the preferred choice for its consistent high performance, but BBMap’s notable resilience under the default setting provides a compelling alternative for scenarios involving high genetic variation. HISAT2 and SubRead offer strong performances in various settings, and even TopHat2 may have its niche applications. The selection of the most appropriate RNA-Seq alignment tool should be informed by the specific needs of the dataset, the research objectives, and the unique characteristics of each tool. Researchers must weigh each tool’s performance and features to secure the most accurate and reliable alignment results for their plant-based genomic studies.

**Figure 3:**
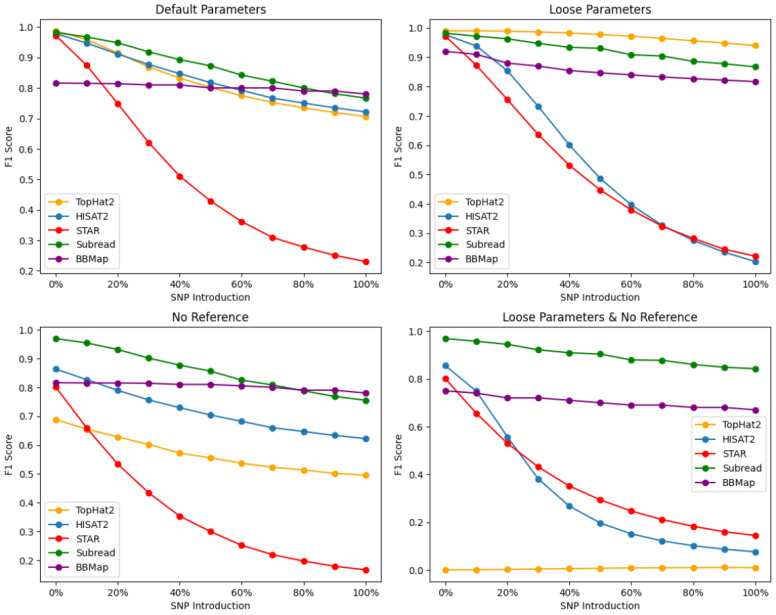
Comparative analysis of junction base-level accuracy (F1-score), produced by RNA-Seq aligners at different parametric settings.

Junction base-level accuracy is crucial for the efficient “spliced” alignment of RNA-Seq reads onto the reference genome, as it measures the recognition of junctions between exons and introns. At the junction base, our analysis reveals a notable decrease in F1-scores compared to the read-base accuracy, as the percentage of SNPs increases for all alignment tools. As depicted in Figure 3, SubRead predominantly outperformed the other alignment tools in terms of junction read-base alignment robustness, except under Loose settings, across all SNP percentages. Under the default settings (Figure 3, top left), the alignment tools are ranked in the following order (in terms of their overall F1-scores): SubRead > HISAT2 > TopHat2 > BBMap > STAR. When employing the Loose settings (Figure 3, top right), the ranking changed to TopHat2 > SubRead > BBMap >HISAT2 > STAR. In the scenario where a reference genome/transcriptome is not provided, the ranking is as follows: SubRead > BBMap > HISAT2 > TopHat2 > STAR. Finally, for both the Loose setting and the absence of a reference genome, the alignment tools are ranked in the following order: SubRead > BBMap > STAR > HISAT2 > TopHat2.

### 4.6. Default Parameters (Junction Base Level)

Under the default parameter setting, SubRead exhibits the strongest performance in junction base-level accuracy, with its F1-scores remaining relatively high across all SNP percentages. This indicates its algorithm’s efficiency in recognizing splice junctions, even amidst genomic variations. HISAT2 and TopHat2 show a gradual decline in their F1-scores with increasing SNPs, suggesting a moderate tolerance to genomic variation. In contrast, STAR experienced the most significant decline (from 0.97 to 0.23 of F1), highlighting potential limitations in its default setting for handling splice junctions with high SNP variations. BBMap, while not outperforming SubRead, still maintains a moderate performance, outshining STAR, which suggests that its approach to junction alignment is more resilient to SNPs than STAR’s, but does not reach the efficiency of SubRead or HISAT2 under the default conditions.

### 4.7. No Reference Parameters (Junction Base Level)

In the absence of a reference genome, SubRead remains the top performer, underscoring its robustness in aligning reads to exon–intron junctions. BBMap takes the second position, outperforming HISAT2 and TopHat2, which indicates BBMap’s ability to handle junction alignments without a reference genome is superior to these tools. HISAT2 still maintains an edge over TopHat2, reflecting improvements inherent in its design. STAR, however, shows the most considerable performance drop (from 0.80 to 0.17 in F1). These observations lend additional support to the notion that STAR’s algorithm encounters difficulties in detecting splicing events as transcriptomic variation intensifies, particularly in the absence of a reference genome.

### 4.8. Loose Parameters (Junction Base Level)

At the loose alignment setting, TopHat2 unexpectedly excels, suggesting that it may handle splice junctions effectively when allowed more flexibility. SubRead’s performance remains robust, indicating its algorithm’s resilience. Despite being in the second rank, SubRead demonstrated a robust performance, which could be attributed to its distinctive seed-and-vote approach conferring a high degree of accuracy, even in the presence of genomic variation.

BBMap falls behind SubRead, indicating that, while it has a flexible approach to junction alignment, it may not be as effective as SubRead under these conditions. HISAT2, despite being TopHat2’s advanced successor, does not perform as well, hinting at possible over-tuning for specificity that does not benefit from looser settings. HISAT2 exhibited superior F1-scores at lower SNP percentages, with the highest of 0.977 at 0% SNP. However, as the SNP percentage escalated, HISAT2’s F1-score incurred a gradual decline, ultimately reaching a minimum of 0.203 at 100% SNP. This pattern implies that HISAT2 is highly sensitive to genomic variations when splice junctions have to be deciphered. 

STAR’s decline is pronounced, indicating that the loose parametric setting negatively impacts its ability to align junctions correctly. The highest F1-score for STAR is 0.972 at 0% SNP and is comparable to that of HISAT2. Nevertheless, at 100% SNP introduction, HISAT2’s F1-score descends to 0.20, which denotes a marginally more pronounced reduction in performance when compared to STAR’s lowest F1-score of 0.22.

### 4.9. Loose and No Reference Parameters (Junction Base Level)

Our analysis revealed that under the Loose and No Reference alignment setting, SubRead emerged as the most robust alignment tool across all SNP percentages, exhibiting F1-scores ranging from 0.969 at 0% SNP to 0.843 at 100% SNP. This observation suggests that SubRead is highly adept at aligning reads to exon–intron junctions without relying on a reference genome, even in the presence of significant genomic variations. BBMap follows, with its performance suggesting it is capable of effective junction alignment without relying on a reference, albeit not as proficient as SubRead. STAR, while ranking below BBMap, shows a better performance than HISAT2 and TopHat2, which may reflect its inherent strengths in managing alignments when both the reference genome and the stringent parameters are absent. HISAT2, as the successor to TopHat2, demonstrated a marked improvement in junction base-level alignment accuracy.

With F1-scores ranging from 0.855 at 0% SNP to 0.076 at 100% SNP, HISAT2 consistently outperformed TopHat2, whose F1-scores were close to 0, regardless of the amount of SNP introduced. This enhancement in performance may be attributed to the more advanced algorithms employed by HISAT2, although these algorithms were outmatched by its predecessor when only Loose settings were applied. In the case of STAR, the F1-scores ranged from 0.801 at 0% SNP to 0.144 at 100% SNP. This indicates a substantial decline in the performance as the SNP percentage increased, particularly when compared to SubRead. Upon comparing TopHat2 and HISAT2, it is evident that HISAT2, as TopHat2’s successor, demonstrates a significant improvement in junction base-level alignment accuracy at the Loose and No Reference setting. 

## 5. Discussion

In this paper, we compared the performance of five popular RNA-Seq alignment tools using the *A. thaliana* genome. As alignment is the first step in an RNA-Seq pipeline, following data pre-processing (quality check, adapter sequence removal, read trimming, etc.), it is highly important for an alignment tool to perform well under a variety of conditions and parameters, as the fidelity of downstream analysis and inference critically depends on accurate read alignment. Oftentimes, genomic data may contain variations such as SNPs, indels, alternative splicing events, and other complexities (e.g., errors arising from sequencing machines) that could pose significant challenges to an alignment algorithm; these may manifest as additional, missing, or mismatched bases in the CIGAR string. An alignment software’s inability to account for these variations or errors could negatively affect downstream analysis. Therefore, we consciously designed our experiment to introduce increasing levels of complexity in the reads—in the form of known SNPs—by creating conditions that test an alignment algorithm’s ability to make accurate decisions. The overarching hypothesis of our study was that the algorithms established as being ‘splice-aware’, namely, HISAT2, STAR, BBMap, and SubRead, would perform better than their more dated counterpart, TopHat2, particularly with junction base-level detection. Furthermore, we hypothesized that, as the alignment methodology drifts away from ‘Default’ parameters, the scoring performance would suffer. By identifying which algorithm performs best with increasingly complex datasets and varying parameter settings, and, importantly, by mapping the strengths and weaknesses of each relative to the others, relevant ‘omics’ research could be conducted using this performance benchmark as a reference, with the awareness of the alignment algorithm that best suits the research problem of interest, especially regarding the investigation of plant transcriptomic data.

Measuring both read base-level and junction base-level accuracy provides valuable insights into each aligner’s efficiency in aligning RNA-Seq reads onto the reference genome in a splice-aware manner. The recognition of the junction between an exon and an intron allows efficient “spliced” alignment, and is quantified through junction accuracy for each aligner. The base-level accuracy accounted for the matches of the read bases to their originating locations in the genome. A study by Musich et al. [19] compared short-read aligners TopHat2, STAR, and HISAT2, and indicated TopHat2 as the lowest performing aligner, with the other aligners achieving ~90% or greater transcriptome coverage. Furthermore, Musich et al. purported the greatest number of unmapped reads as belonging to TopHat2′s output. With TopHat2′s discontinued support and supersession by HISAT2, it is unsurprising that it performs least favorably in the previous studies, as well our benchmarks. HISAT2 uses a hierarchical indexing scheme and incorporates local alignment to enhance splice junction detection. This approach might provide a slightly higher degree of sensitivity and precision in detecting splice junctions in the presence of genomic variations. On the other hand, STAR uses a seed-and-extend strategy, which may be more vulnerable to the effects of increased genomic variation. In particular, the STAR’s algorithm might struggle to detect splicing events as the level of transcriptomic variation increases, leading to the observed decrease in performance. Note that, by citation count, TopHat2 is the second most popular alignment tool of all those chosen, with >11,000 citations at the time of this writing, and is still being used and cited by many researchers. STAR is the most popular, with >23,000 citations, HISAT2 with >2300 citations, Subread with >1900 citations, and BBMap with >1300 citations; all indicative of the relative popularity of these alignment tools.

### 5.1. Read Base-Level Analysis 

In the comparative assessment of the alignment tools at their default parameter settings, STAR yielded the best overall performance, with the overall accuracy F1 > 0.96 in the read base alignment evaluation. This performance trend was maintained at the other settings as well (i.e., Loose, No Reference, and Loose Without Reference). STAR’s high performance could be attributed to its efficient maximal mappable prefix (MMP) search, which enables mismatches and indels to be detected by taking both forward and reverse directions of a read to locate anchors and increase the mapping sensitivity of reads with higher error rates [14]. STAR’s superior performance could also be attributed to its stitching procedure, which allows its algorithm to align reads with high error rates (mismatches, indels) without the need for seeds to precisely match to a region in the reference genome. 

BBMap also maintained high F1-scores across a broad range of SNP introductions, showcasing its robustness against polymorphic variations. As expected, TopHat2 was outperformed by other alignment methods, yet it produced an F1-score of over 0.84 for base-level accuracy under all different test conditions. Overall, each algorithm performed reasonably well, even after the introduction of higher levels of SNP content. Our results for default parameters were fairly consistent with those from previous benchmarks conducted with HISAT2, STAR, and TopHat2 using *Plasmodium falciparum* [20].

### 5.2. Junction Base-Level Analysis

As compared to base-level accuracy scores, the junction base-level results were far more variable. Compared with other algorithms at the default parameter settings, STAR had the worst overall performance (F1-score), although it displayed its best performance at the base-level resolution. STAR’s performance at its default alignment parameters was approximately 42% lower than SubRead, which was the best aligner for junction base-level accuracy at its default setting. This result is particularly intriguing as STAR is a splice-aware algorithm. SubRead was the best performing algorithm at a junction base-level resolution under all conditions, except for Loose alignments, where TopHat2 outperformed others. BBMap’s performance did not match that of SubRead or TopHat2 in the Loose conditions, indicating potential areas where its junction alignment strategies could be enhanced. 

High variability in the performance of aligners was observed at the Loose and No Reference setting. At this setting of No Reference genome and a lowered confidence threshold for alignment, TopHat2′s algorithm was ineffective at predicting junction bases. TopHat2′s spliced alignment algorithm is able to identify splice sites for known transcripts; however, without a reference genome, its algorithm fails to predict junction bases in our experiments. In the TopHat2’s original study, the algorithm was tested using reads with relatively small indels, ranging from 1 to 3 bp. Our SNP introducer incorporated known polymorphisms from TAIR10, which the algorithm likely could not recognize in the absence of a reference genome. On the other hand, at this setting, the best performer SubRead had an F1-score of 0.90. BBMap’s performance, surpassing that of HISAT2 and TopHat2, suggested its effective approach to junction alignment, independent of a reference genome. The adaptability of SubRead and BBMap in these challenging conditions highlights their utility in studies where reference data may be incomplete or highly variable. BBMap’s moderate performance, though not leading, was significant enough to position it as a reliable tool in the suite of RNA-Seq aligners, especially for its ability to handle junctions without reference guidance.

## 6. Conclusions

Most researchers will likely use aligners at their default parameter setting for the alignment of RNA-Seq data. Our results highlight the strengths and weaknesses of different alignment algorithms at their default and other settings. This is important as the fidelity of downstream analyses depends on robust read alignment. Benchmarking studies, such as ours, inform the users about alignment tools that are robust and optimized for alignment under specific conditions. Our benchmark analysis revealed that the most popular RNA-Seq aligners generally perform quite well at the base-level resolution for plant research; however, a user needs to be more cautious and selective in choosing an RNA-Seq algorithm if higher junction base-level accuracy is desired. SubRead’s alignment algorithm appears to be the most promising overall, and is recommended for experiments requiring a high junction base-level accuracy. Although the scope of this study was restricted to assessment with respect to the *Arabidopsis thaliana* genome─ a diploid genome, perhaps less complex than many other plant genomes in various aspects─ interesting insights, hitherto unknown, into alignment tools used frequently in the *A. thaliana* transcriptomic studies were gained from our benchmarking study. Future studies could focus on assessing the aligners under more complex conditions, such as on polyploid plant genomes, and furthermore, on plant genomes with a high repeat content, as well as on those with high heterozygosity rates. 

## Data Availability

The data and code presented in this study are openly available here: https://github.com/tallon-research/RNA-Seq-Alignment-Benchmark-for-Arabidopsis (accessed on 16 January 2024). Publicly available datasets were analyzed in this study, which can be found here: https://www.arabidopsis.org/download/index.jsp (accessed on 16 January 2024).
